# The damping properties of the foam-filled shaft of primary feathers of the pigeon *Columba livia*

**DOI:** 10.1007/s00114-021-01773-7

**Published:** 2021-12-03

**Authors:** K. Deng, A. Kovalev, H. Rajabi, C. F. Schaber, Z. D. Dai, S. N. Gorb

**Affiliations:** 1 Functional Morphology and Biomechanics, Institute of Zoology, Kiel University, Kiel, Germany; 2 School of Engineering, London South Bank University, London, England; 3 Institute of Bioinspired Structure and Surface Engineering, Nanjing University of Aeronautics and Astronautics, Nanjing, China

**Keywords:** Bird, Vibration, Biomechanics, Laser Doppler vibrometry, Frequency, Flight

## Abstract

The avian feather combines mechanical properties of robustness and flexibility while maintaining a low weight. Under periodic and random dynamic loading, the feathers sustain bending forces and vibrations during flight. Excessive vibrations can increase noise, energy consumption, and negatively impact flight stability. However, damping can alter the system response, and result in increased stability and reduced noise. Although the structure of feathers has already been studied, little is known about their damping properties. In particular, the link between the structure of shafts and their damping is unknown. This study aims at understanding the structure-damping relationship of the shafts. For this purpose, laser Doppler vibrometry (LDV) was used to measure the damping properties of the feather shaft in three segments selected from the base, middle, and tip. A combination of scanning electron microscopy (SEM) and micro-computed tomography (µCT) was used to investigate the gradient microstructure of the shaft. The results showed the presence of two fundamental vibration modes, when mechanically excited in the horizontal and vertical directions. It was also found that the base and middle parts of the shaft have higher damping ratios than the tip, which could be attributed to their larger foam cells, higher foam/cortex ratio, and higher percentage of foam. This study provides the first indication of graded damping properties in feathers.

## Introduction

Flight has given the animals survival advantages, by providing efficient locomotion and various habitats (Clark 2013). In avians, wings consisted of the flight feathers needed to sustain the dynamic force and vibration during the flapping (Greenewalt 1960). Excessive vibrations can lead to more consumed energy, noise, and negatively impact flight stability. It is reasonable to hypothesize that feathers of wings have undergone adaptations to develop several mechanisms to mitigate vibrations.

**Fig. 1:**
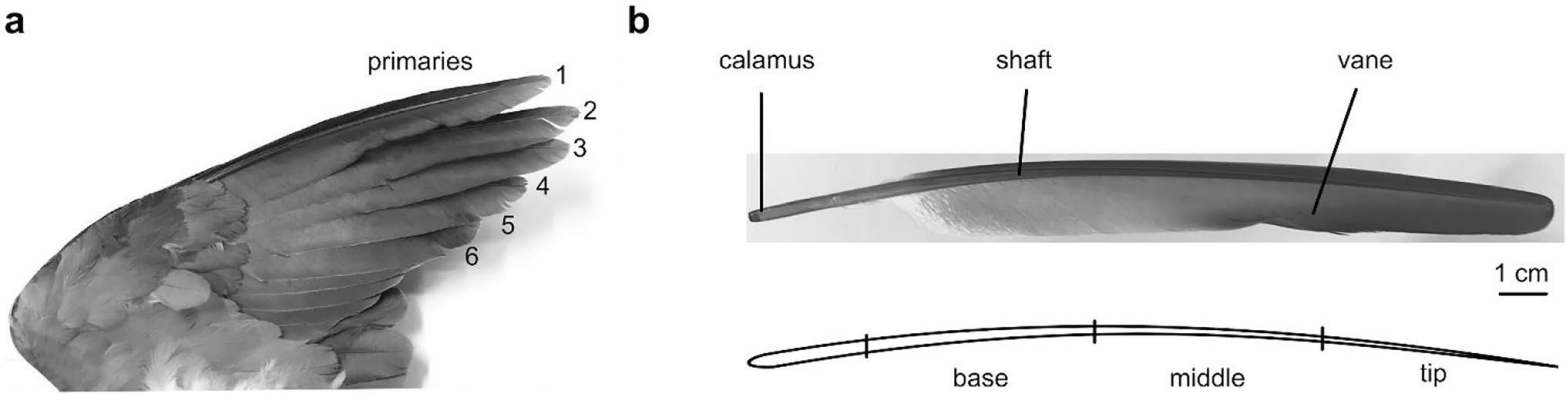
Primary feathers of the rock pigeon *Columba livia* used in the study (ventral view). (a) Location of primary feathers. (b) An intact primary feather. (b) Scheme of the shaft segments

Feathers originated in theropod dinosaurs as simple filaments of varying lengths and diameters (Clark 2013). Feathers of modern birds, in contrast, evolved into complex hierarchically organized epidermal structures (Prum 1999; Bragulla and Hirschberg 2003). During feather evolution, pennaceous vanes for flight and display, and fluffy plumulaceous branches evolved for thermoregulation (Chang et al. 2019). Typical bird flight feathers consist of a shaft and two vanes (Fig. 1). The shaft is composed of a rigid outer shell and an inner foam-like structure, which shows a longitudinal gradient of cell sizes (Bonser and Purslow 1995). The vane consists of numerous barbs aligned parallel to each other, but at some angle to the shaft (Prum 1999; Clark 2013). The barbs are loosely connected by the elaborate system of hooklets and, when separated by an external force, they can easily re-establish their connections (Ennos et al. 1995; Butler and Johnson 2004; Kovalev et al. 2013; Gao et al. 2013; Chen et al. 2016; Sullivan et al. 2016).

Similar to other flyers such as dragonfly and damselfly, oscillations might negatively influence the stability (Rajabi et al. 2011, 2016). Hence, damping or energy dissipation is present in such a dynamic system. Damping alters the response of the system and results in desirable effects, such as stability and enhanced controllability (De Silva 1999). Therefore, it is plausible to hypothesize that there are structural mechanisms that enhance the damping properties of feathers and, thereby, reduce unwanted oscillations during flight.

Concerning the feather shaft, damping-related loss factors (tan δ) of swan, eagle, owl, and pigeon have been previously measured (Bonser and Purslow 1995; Gao et al. 2014). Loss factor, the ratio of loss modulus to storage modulus, is a parameter that presents the viscoelasticity and the damping capacity. For a small damping ratio, the loss factor is twice the damping ratio, tan δ = 2 ζ (Hansen 2018). A low damping ratio ranging from 0.015 to 0.035 has been revealed for bending of the swan feathers (Bonser and Purslow 1995), while the damping ratio of owl flight shafts has been estimated to be 0.804 ± 0.119, which is larger than those of gold eagle 0.543 ± 0.037 and pigeon 0.448 ± 0.041 (Gao et al. 2014). However, a systematic understanding of the relationship between gradient structure and damping property of feathers is still far from being achieved.

Feather, as a mechanical structure, is lightweight and combines this feature with specific robustness and flexibility (Raspet 1960; Prum 1999). This combination is supposed to be the result of the specific hierarchical organization at the macro-, micro-, and nano-scales (Yang et al. 2013). It has been suggested that the mechanical properties of the shaft are mainly dominated by the properties of the outer cortex (Purslow and Vincent 1978; Bachman et al. 2012). Inside of the cortex, foams are known to be a design strategy to enhance mechanical stability by energy absorption (Bonser and Purslow 1995; Banhart and Baumeister 1998; Bonser 2001; Gao et al. 2014).

In this study, we aimed to understand the contribution of the gradient foam-filled shaft to the damping behavior of primaries. Measurements, using laser Doppler vibrometry (LDV), were performed on specimens selected from different shaft regions, to study variations of damping properties along the shafts. A combination of scanning electron microscopy (SEM) and micro-computed tomography (µCT) was used to explore the correlation between the shaft morphology, its microstructure, and measured damping properties.

## Materials and methods

### Sample preparation

Adult female pigeons *Columba livia* were taken from the collection of the Zoological Institute of Kiel University, Germany. Preliminary experiments showed the presence of more than one oscillatory mode in vibrations of deflected feather shafts after release. Hence, to facilitate the data analysis, we introduced a simple manipulation to the system: the oscillations were studied with a mass attached to the free distal end of each segment. In this way, only two resonance frequencies were observed in the deflected shafts after release.

**Fig. 2:**
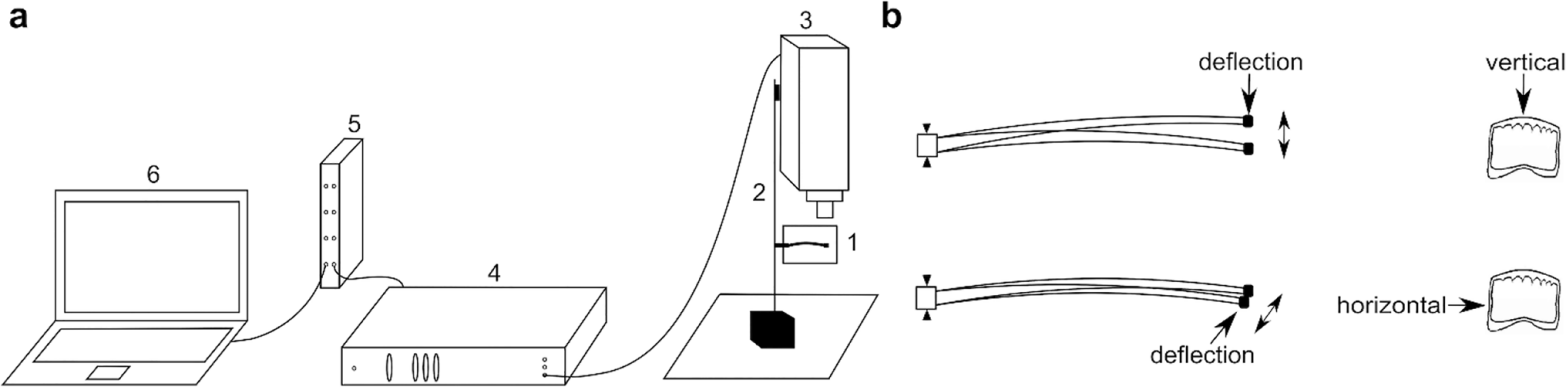
Damping measurements. The oscillation of the shaft specimen was measured using a velocity-sensitive laser Doppler vibrometry (LDV). (a) Experimental setup: feather specimen (1), support (2), objective lens (3), video monitor (4), waveform analysis equipment (5), and software control (6). (b) Excitation of oscillations of the feather shaft segments by its tip deflection and abrupt release vertically (upper panel) perpendicularly to the vane, and horizontally in the plane of the vane (lower panel)

For further studies, the primary flight feathers were cut out from the wing (Fig. 1a). Primary feathers 1–6 (165–180-mm length) were sectioned, and 18 shaft specimens were used for damping tests. Vanes were cut from the feathers using a razor blade. The shaft was transversely sectioned in 50-mm-long intervals from the tip, dividing the shaft into three parts (Fig. 1b). The most basal part of the shaft, the calamus, was excluded from the experiments. The basal part of the specimens was embedded in epoxy resin and then mechanically fixed (Fig. 2b, black triangles). Primary feather 7 (160-mm length) was used for μCT analysis.

### Damping test

In this study, damping tests were performed under room temperature 20–22 °C and humidity 40–45%. Damping tests were conducted on the basal, middle, and tip segments of the shaft. The test specimens were mechanically fixed at their basal parts in a 3D micro-manipulator. To reduce the oscillation decay and to simplify the frequency analysis, as mentioned earlier, an extra mass (of 2.015 g) was attached at the free distal end of the specimens. The specimens were excited to oscillate by releasing from an initial deflection of 15 mm. The oscillations were excited in the vertical (perpendicular to the vane) and horizontal (parallel to the vane) directions (Fig. 2b). The decaying oscillation velocity was measured using a laser Doppler vibrometer (Polytec, Waldbronn, Germany) (Fig. 2a). The experimental setup including the mechanical supporters holding the feather specimen and the laser displacement sensor is shown in Fig. 2a. The oscillation velocity was recorded with a sampling rate of 5 kHz and observed in one direction, perpendicular or parallel to the vane plane. The original experimental recordings were then converted from the analysis software package Spike2 (CED, version 4.24) (Cambridge Electronic Design Limited, Cambridge, England) to the software Origin Pro 8.0 (OriginLab, MA, USA).

### Feather morphology

An SEM Hitachi S-4800 (Hitachi High-Technology, Tokyo, Japan) was used at an acceleration voltage of 3 kV to study the feather microstructure at different levels of the shaft. Dry samples were attached to aluminum stubs using adhesive tape and then sputter-coated with 8-nm gold–palladium using a Leica EM SCD 500 (Leica Microsystems, Mannheim, Germany).

Skyscan 1172 µCT (Bruker microCT, Kontich, Belgium) was used for three-dimensional scanning of shaft specimens (1 primary feather, 8 segments). The X-ray voltage and current were set to 40 kV and 250 μA, respectively. The NRecon software (SkyScan, Kontich, Belgium) was used to reconstruct structures from the 3D data of µCT scans. The segmentation was performed using the image analysis software Amira (FEI Visualization Sciences Group, Bordeaux, France).

### Fitting of oscillation curves

In this study, the equation of under-damped oscillations was employed to describe the damping behavior of the shaft. For shaft segments with a terminal mass, the oscillations behavior is under-damped. The motion of the shaft end can be described by the following equation (Rao 2010):

1 $$X=A{e}^{-\beta t} \mathrm{cos}(\omega t+\varphi )$$

where *X* and *t* are the displacement and time, *β* is the damping coefficient, *A* is the initial amplitude, *ω* is the circular frequency, and *φ* is the phase angle. The presence of two vibration modes (frequencies) was observed in experiments on oscillating shaft segments. Therefore, a second term for the second vibration mode as well as an offset, *A*_0_, was added to Eq. 2:

2 $$X={A}_{0}+{A}_{1} {e}^{-{\beta }_{1}t}\mathrm{cos}\left({\omega }_{1}t+{\varphi }_{1}\right)+{A}_{2} {e}^{-{\beta }_{2}t}\mathrm{cos}\left({\omega }_{2}t+{\varphi }_{2}\right)$$

where indices 1 and 2 refer to the first and second vibration modes. To perform the fit of experimental curves with Eq. 2, the initial values of the fit parameters were determined as follows. Fast Fourier transformation (FFT) was employed to estimate the first (*ω*_*1*_) and second (*ω2*) frequencies and initial phases *φ*_1_ and *φ*_2_ of the shaft segments. Initial amplitudes *A*_1_ and *A*_2_ were taken equal to half of the peak value in the experimental velocity–time curve.

However, the damping coefficient, *β*, is related to the frequency (Beards 1996), while the damping ratio, *ζ*, is frequency independent. The damping ratio was calculated using Eq. 3:

3 $$\zeta =\beta /\omega$$

To compare our results with those previously reported in the reference (Bonser and Purslow 1995; Gao et al. 2014), the damping ratios will be further presented.

## Results and discussion

### Damping properties of shaft segments

**Fig. 3:**
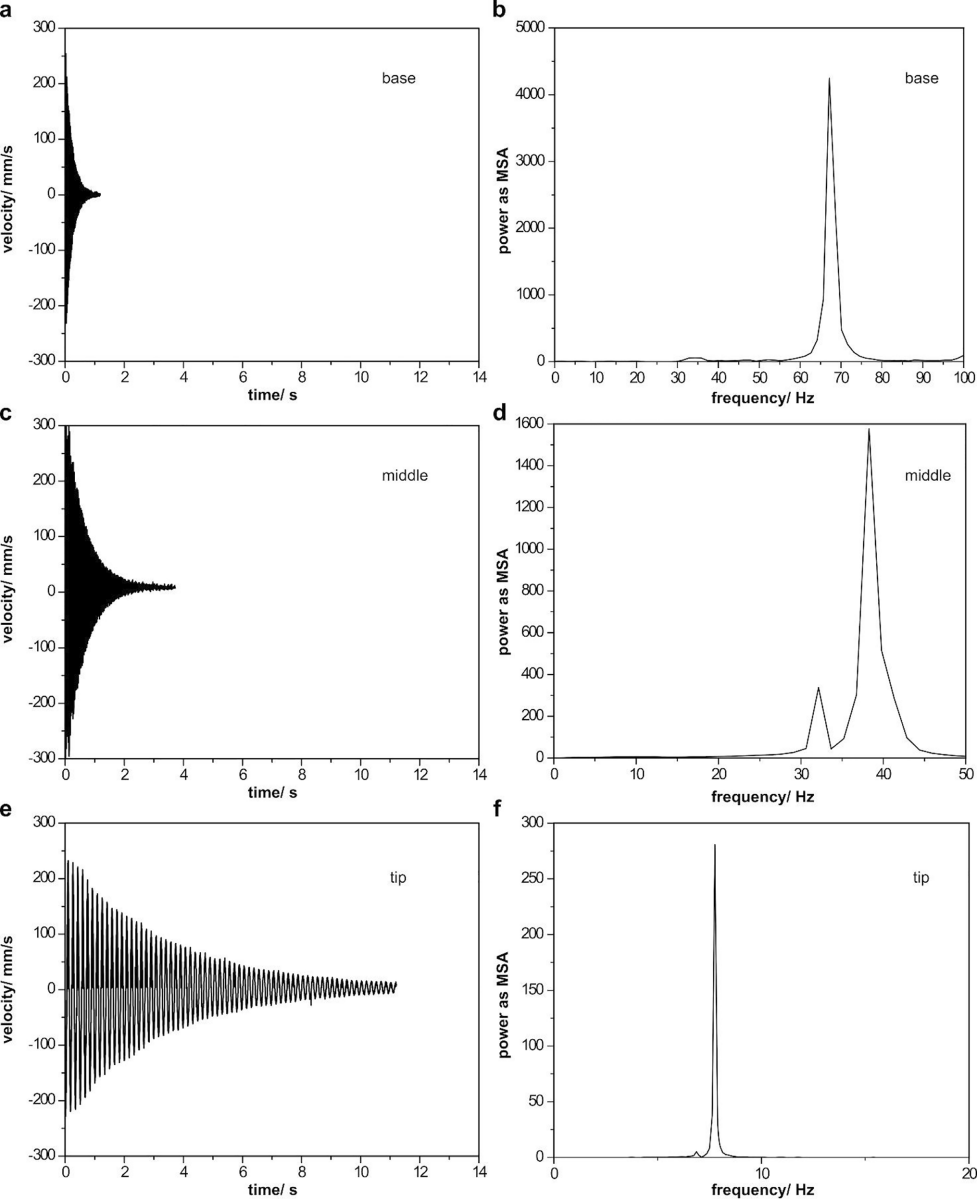
Typical oscillation curves for the base (a), middle (c), and tip (e) segments of the feather shaft with corresponding power spectra for the base (b), middle (d), and tip (f) segments representing velocity mean square amplitude (MSA)

After deflecting the free end of the shaft specimens, they started to oscillate. The velocity of the oscillations decreased over time until they stopped. The oscillations of all shaft segments showed typical characteristics of an under-damped regime (Fig. 3). The decay times of the shaft specimens are roughly 1 s at the base, 3 s in the middle, and 12 s at the tip. The power spectra of the velocity-decay time curves calculated using fast Fourier transform (FFT) are illustrated in Fig. 3b, d, f. Two frequencies were observed in all segments: 54.66 ± 17.37 Hz and 66.62 ± 9.91 Hz at the base, 29.46 ± 1.75 Hz and 38.14 ± 4.16 Hz in the middle, 6.46 ± 0.40 Hz and 8.16 ± 1.41 Hz at the tip of the shaft.

**Fig. 4:**
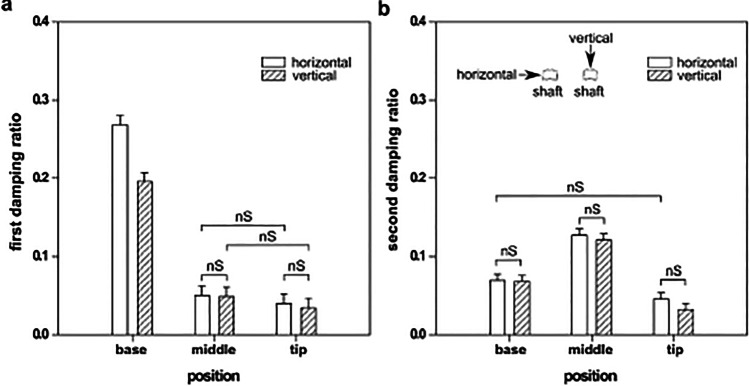
Damping ratios of (a) the 1st (lower) frequency and (b) the 2nd (higher) frequency oscillations in different segments of the feather shaft under vertical and horizontal deflections. Measurements were performed for 6 feather shafts, 18 segments, and repeated three times. nS indicates no significant difference according to the results of the two-way ANOVA test

The comparison of damping ratios of the shaft segments in the horizontal and vertical deflections (as Fig. 2b) is illustrated in Fig. 4. Under both deflections, the damping ratios of the 1st mode at the base are higher than those in the middle and tip segments. In the 2nd mode, in contrast, the maximum values of the damping ratios were observed in the middle segment. The damping ratio in the horizontal direction was higher than that in the vertical direction.

As seen, the damping ratios of primary feather shafts from the pigeon range from 0.268 to 0.034 in the 1st mode, and 0.127 to 0.032 in the 2nd mode (Fig. 4). Our results are lower than the previous data obtained from a uniaxial tensile test (Gao et al. 2014), where the of the pigeon shafts was found to be 0.448. This difference can be due to the use of different specimens and measurement techniques.

### Feather shaft morphology

#### Cortex morphology

**Fig. 5:**
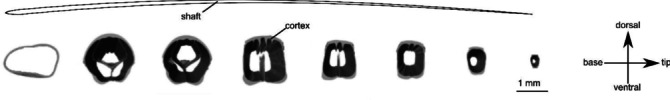
Cross-sections of shaft segments were obtained from the µCT scans. Segments were taken from the primary feather No. 7 (separated into 8 segments). The morphology of the cortex within the shaft is shown from the calamus to the distal tip. The shape and diameter changes of the shaft are visible

The three-dimensional reconstructions based on μCT data show structural variations of the cortex (Fig. 5). The diameter and the cross-sectional area decrease continuously from the feather base towards its tip. The shape of the cortex varies from an almost circular shape at the base, to a rectangle in the middle, and finally to an ellipse at the tip. It is known that, given the same cross-sectional area, square cross-sections show higher bending rigidity and are superior in maintaining the original shape under deformation, in comparison to circular sections, which become oval-shaped upon flexing (Prum and Williamson 2001; Wang and Meyers 2016). The square shape with a high area moment of inertia, using a larger amount of material, would be mechanically favorable in bending, but also heavier. Taking into account that feathers are anchored to the bone, the circular base has grown from cylindrical feather follicles perhaps providing better mechanical connectivity (Prum and Williamson 2001; Wang and Meyers 2016). However, to achieve improved resistance to forces applied to the feather during flight at a minimal weight, the cross-sectional shape of the feather transforms to a square in the middle, and then to a more elliptical shape at the tip.

In the interior dorsal surface of the cortex, ridges are running from 1/4 to 3/4 of the feather length (Fig. 5). At the base of the shaft, next to the calamus, two dorsal ridges are present. In the middle of the feather, four ridges appear. The ridges transform into a serrated structure and disappear at the tip. The ridges are likely to contribute to a tight bonding between the stiff cortex and flexible medulla by increasing the interfacial area (Zhang et al. 2012).

**Fig. 6:**
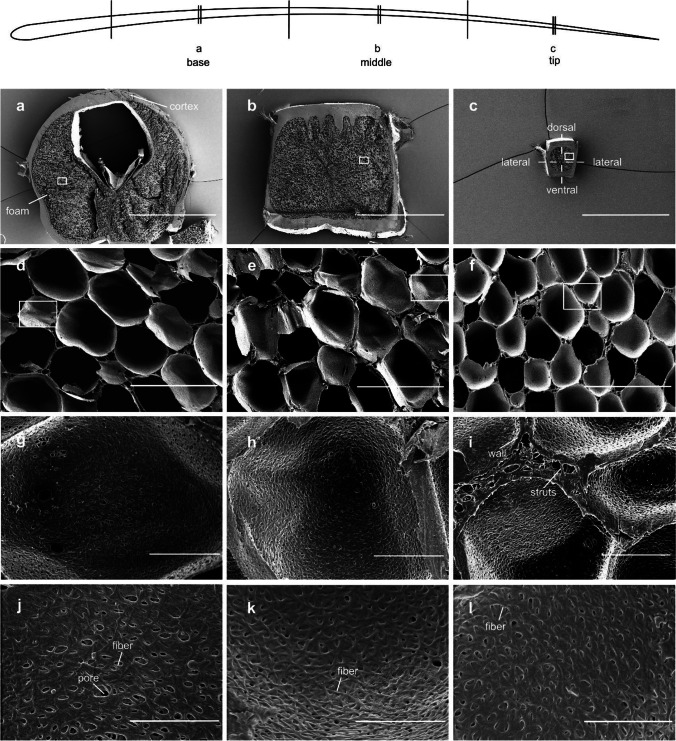
SEM images of transverse sections of shaft segments taken at 150–100 mm (base), 100–50 mm (middle), and 50–0 mm (tip) away from the tip. (a, d, g, j) Basal region. (b, e, h, k) Middle region. (c, f, i, l) Tip of the shaft. (a–c) Scale bars: 1 mm. (d–f) Scale bars: 50 μm. (g–i) Scale bars: 10 μm. (j–l) Scale bars: 5 μm

Along the shaft, the thickness of the cortex changes (Fig. 6). This decreasing thickness may enhance the damping performance, as shown for the stick insect antenna with a longitudinal thickness gradient (Rajabi et al. 2011). The feather cortex has also a radial gradient of the thickness in cross-section. The cortex is thicker on the dorsal and ventral sides if compared with the lateral sides (Table 1). This difference could be explained by a potential need to resist higher levels of stress experienced by the dorsal and ventral bending during flight (Sullivan et al. 2016). The mechanical behavior of the shaft is mainly determined by the cortex (Bonser and Purslow 1995; Lingham-Soliar et al. 2010; Liu et al. 2015). The flexural stiffness depends on the second moment of the area and Young’s modulus, which in turn depends on the keratin bundles formed in the cortex (Lingham-Soliar et al. 2010; Liu et al. 2015).

**Table 1:** The thickness of the cortex in three segments of feather shafts

Location	Dorsal thickness/mm	Ventral thickness/mm	Lateral thickness/mm
Base	0.135 ± 0.021	0.153 ± 0.013	0.0718 ± 0.003
Middle	0.124 ± 0.006	0.147 ± 0.007	0.0325 ± 0.005
Tip	0.103 ± 0.011	0.107 ± 0.014	0.045 ± 0.003

#### Medullary foam structure

**Fig. 7:**
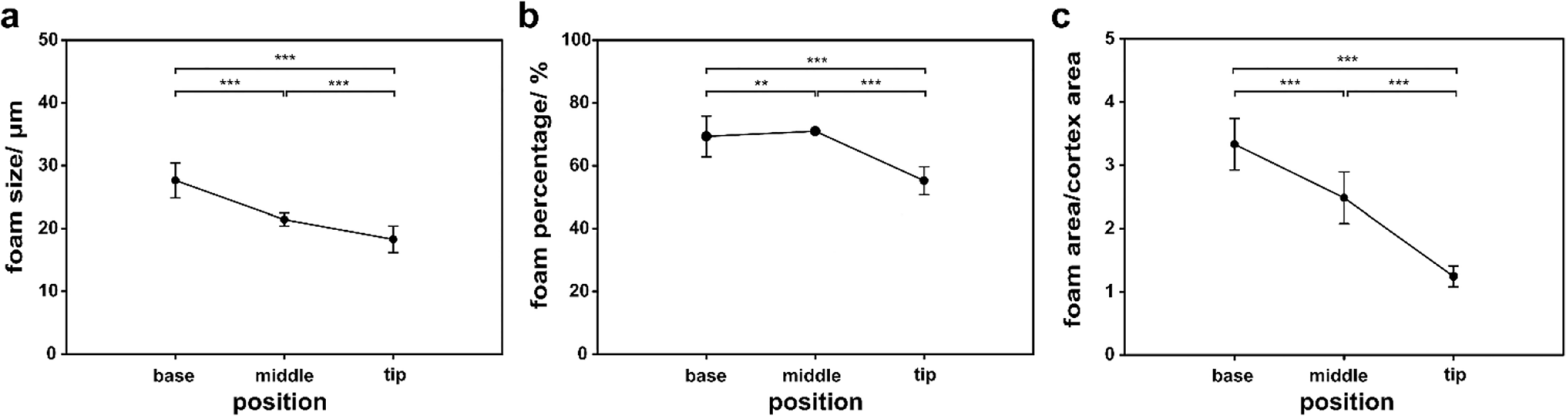
The foam morphology and relationship between medulla and cortex. (a) Size of foam cells in the base, middle, and tip regions. (b) The relationship between the foam area and shaft segments. (c) The ratio between the medullar foam area and the cortex area along the shaft

The shaft is filled with foam cells. The shape and morphology of foam cells demonstrate a gradient (Figs. 6 and 7). At the base, the foam cells are relatively large with a diameter of about 27.09 ± 1.08 µm. Their size decreases to 21.35 ± 0.40 µm in the middle, and to 18.21 ± 0.46 µm at the tip (a decrease of ~ 15% and ~ 32%, respectively). The cells are polyhedrally or spherically shaped, and the walls (membranes) are very thin (~ 0.08-µm thickness). The SEM analysis also revealed that the walls and struts are closely packed in the septa. The fragmentary or columnar struts interconnect the foam cells and strengthen the cell interfaces. The continuous wall likely serves as the primary load supporter increasing the stability of the foams. The cell walls are made of irregularly orientated and curved weaving fibers (Fig. 6j–l). The fibers of cells merge with the fibrils composing the cortex, which enhance the mechanical stiffness of the medulla foam.

Bird wings in general and feathers, in particular, are simultaneously robust and light enough to meet the mechanical demands to generate aerodynamic forces (Pennycuick 1968). The shaft is an important part, which consists of medullary foam and cortex (Sullivan et al. 2017). The tubular structure is perhaps the simplest solution to maximize the stiffness at a minimal weight (Bonser 2001). However, a tubular structure is likely to fail by buckling (Bonser 2001). The foam-like lightweight medulla within the shaft is much less stiff than the cortex but also contributes to the mechanical performance of the shaft (Purslow and Vincent 1978; Bonser 2001; Lingham-Soliar et al. 2010). It has been previously shown that the removal of the medulla reduces the stiffness of the shafts by 16% (Bonser 2001). The estimated Young’s modulus of the foam showed considerable variation, ranging from 5 MPa to 1.58 GPa (Bonser 2001). The foam is expected to delay the onset of buckling under compression (Bonser 2001) and bending (Purslow and Vincent 1978) by transferring the stress from the cortical layer to medulla foams. The foams are also likely to dissipate energy and, thereby, contribute to the damping properties of the feathers.

### Effect of cortex and gradient foam structure

Similar to other dynamic systems, unwanted oscillations could reduce the aerodynamic performance in flying animals (Rajabi et al. 2011, 2016; Alexander 2015). It is known that bird wings have vibration-sensitive mechanoreceptors, either at the follicle or Herbst corpuscles (Bruecker et al. 2016). Apart from the active control of energy-consuming muscles and neural systems (Alexander 2015), the feathers with damping capability could help to passively minimize undesirable vibrations. The flight feathers are anchored to the ulna and metacarpals/phalanges, where they are supplemented with muscles and sensors (Kondo et al. 2018). The higher damped base of the shaft assures the stability of the wing bone under oscillations.

A gradient of damping properties is present in the shaft. The observed structural gradients, both of the cortex wall and foams, are assumed to be responsible for the different damping properties of the three segments of the shaft. The cortex consists of multiple layers of aligned collagen fibers (Liu et al. 2015). It is plausible to assume that friction between these fibers and layers may contribute to the damping properties. The presence of ridges increases the cortex-foam interface and therein leads to increased friction between them. The filled medulla, which occupies a large part of the shaft’s cross-section, is assumed to strongly contribute to the damping properties of the shaft. The damping ratio following the size of foam cell and area ratio of the foam/cortex change in the 1st vibration mode (Figs. 4a and 7a, c). It means that the larger foam cells and the higher area ratio between foam and cortex are, the higher is the damping ratio. The higher percentage of foam structure is proportional to the higher damping ratio in the 2nd vibration mode (Figs. 4b and 7b).

Bending, shear, and buckling are the main deformation modes in the foam structure (Vincent and Owers 1986; Gibson et al. 1989; Gibson and Ashby 1997). During shaft bending, the cells situated remote from the central axis experience the highest stress (Nillsson and Nillsson 2002). At small strains, the walls elastically bend, the foam cells/struts undergo the shear deformation, and cell walls may experience no stretching. At large strains, beyond a critical value, the cells may collapse by elastic buckling and plastic yielding or even fracture. At higher strains, the cells collapse and undergo densification with rapidly increasing stiffness (Triantafillou et al. 1989; Gibson and Ashby 1997). Several mechanisms participate in the energy dissipation in foams (Schwaber 1973). Foams dissipate deformation energy across layers, reduce possible crack propagation, and delay the local buckling (Vincent and Owers 1986; Bonser 2001). The cell walls which are composed of curved weaving fibrils absorb energy known as intrinsic damping (Andersson et al. 2010). The fiber-composite cells dissipate energy by fiber pullout (Gibson and Ashby 1997). The difference under the two deflections may depend on the anisotropy of the cortex and foam-like medulla structure. The cell sizes, relative density, and anisotropy have proven to be important parameters governing the mechanical properties of the foam (Gibson and Ashby 1997).

Consequently, the combination of the cortex and the gradient foam makes the feather shaft a lightweight structure with varied damping properties. The higher damped base anchored to the bone assures the wing stability under oscillations. To decay the oscillations in flapping, avian feathers have reached certain levels of damping due to their specialized macrostructures and microstructures.

## Conclusions

The feather shaft represents natural gradient material with damping properties that have been previously oversimplified in the literature. This paper provides the first indication of the graded damping properties of the shaft in feathers. Under horizontal and vertical deflections, the damping ratios at the base are highest in the 1st mode, and the maximum values were observed in the middle of the shaft in the 2nd mode. The higher damping of the base and the middle of the shaft close to the bone assures the stability of the wing under oscillations. SEM and µCT analyses were used to investigate the gradient microstructure of the shaft. The closed-cell medulla is based on curved weaving fibrils with hierarchical levels of porosity. The energy dissipation of the shaft is likely due to the deformations and the densification of foam cells. The higher damping properties are influenced by the larger foam cells, higher area percentage of the foam, and higher area ratio between the foam and cortex. This study represents the first step in exploring damping mechanisms in bird feathers. It also offers an interesting design that may inspire technical structures with adjustable damping properties.
